# Establishment of High-Throughput Screening Assay using 384-Well Plate for Identification of Potent Antioxidants from Malaysian Local Plants Repository and Phytochemical Profile of *Tetracera Scandens*

**DOI:** 10.21315/tlsr2025.36.2.2

**Published:** 2025-07-31

**Authors:** Amyra Amat Sain, Azimah Amanah, Mohd Hasnan Mohd Noor, Wai Kwan Lau, Olalere Olusegun Abayomi, Zafarina Zainuddin

**Affiliations:** 1 Malaysian Institute of Pharmaceuticals and Nutraceuticals (IPharm), National Institutes of Biotechnology Malaysia, Blok 5-A, Halaman Bukit Gambir,11700 Gelugor, Pulau Pinang, Malaysia; 2 Analytical Biochemistry Research Centre (ABrC), Universiti Sains Malaysia, 11800 USM Pulau Pinang, Malaysia

**Keywords:** High-Throughput Screening (HTS) Assay, Plant Extracts, Antioxidants, Secondary Metabolites, Asai Penabiran Celusan Tinggi, Ekstrak Tumbuhan, Antioksidan, Metabolit Sekunder (Fitokimia)

## Abstract

The increasing prevalence of chronic diseases and oxidative stress-related conditions has led to a growing interest in natural products with potent antioxidant properties. High-throughput screening (HTS) assays offer a promising solution for the rapid evaluation of numerous samples. This study aims to establish a robust HTS assay using the 2-Diphenyl-1-Picrylhydrazyl (DPPH) method to identify natural products with significant antioxidant capabilities, ensuring their safety profiles through cytotoxicity screening and phytochemical profiling. An automated liquid handler with the usage of a 384-well assay plate was employed in optimising and validating the 2,2-diphenyl-1-picrylhydrazyl (DPPH)-HTS assay by calculating the metrics. The HTS metrics include Z-prime (Z’) values of 0.72 and 0.63, signal to background (S/B) ratios of 3.54 and 9.02, and coefficient of variation percentages (%C/V) of 4.25 and 6.49 for each primary and secondary screenings, respectively. These values indicated that the HTS assay was excellent. By using optimised HTS-DPPH assay programme, a total of 363 plant extracts were screened and 58 (16%) were found to have potent antioxidant activity (a ‘yes’ score and EC_50_ < 50 μg/mL). Out of these 363 extracts, only 80 plants were identified with herbarium and the screening results revealed that *Tetracera scandens* along with other 11 plants have potent antioxidant activities. *T. scandens* was the most potent and the methanol extract from its leaves recorded an EC_50_ value of 13.041± 0.82 μg/mL. However, the aqueous extract of *T. scandens* leaves (TSLAE) was selected to allow any possibilities of using it for traditional herbal preparation. TSLAE presented an EC_50_ value of 13.76 ± 4.50 μg/mL in the DPPH assay and was non-toxic towards normal cells with an IC_50_ value of > 100 μg/mL. Secondary metabolites with promising antioxidant potentials were successfully identified using LC/MS and the library, which were mainly flavonoids (37.5%), phenolics (8.9%) and polyphenols (8.9%). HTS-DPPH is a robust and rapid technique for screening of antioxidative substances in plant extracts. The hit is non-toxic in vitro and rich in secondary metabolites that contribute to antioxidant activity.

## Abstrak

Kajian ini bertujuan untuk membangunkan asai penabiran celusan tinggi (HTS) untuk menyaring produk semulajadi (NP) bagi agen anti-oksidatif, kajian sitotoksisiti dan pemprofilan fitokimia. Pengendali cecair automatik dengan penggunaan plat ujian 384-telaga telah digunakan dalam mengoptimakan dan mengesahkan ujian 2-Diphenyl-1-Picrylhydrazyl (DPPH)-HTS berdasarkan kiraan metrik HTS. Metrik HTS yang diperolehi termasuk nilai Z-prima atau faktor Z (Z’) 0.72 dan 0.63, nisbah isyarat-kepada-latar belakang (S/B) 3.54 dan 9.02, dan peratusan pekali variasi (%C/V) sebanyak 4.25 dan 6.49 untuk setiap saringan primer dan sekunder. Nilai ini menunjukkan bahawa cerakin HTS adalah sangat baik. Dengan menggunakan program ujian HTS-DPPH yang dioptimakan, sejumlah 363 ekstrak tumbuhan telah disaring dan 58 (16%) didapati mempunyai aktiviti antioksidan yang kuat (skor ‘ya’ dan EC_50_ < 50 μg/mL). Daripada 363 ekstrak ini, hanya 80 tumbuhan yang dikenal pasti dengan herbarium dan hasil saringan mendedahkan bahawa *Tetracera scandens* dan 11 tumbuhan lain mempunyai aktiviti antioksidan yang kuat. *T. scandens* adalah antioksidan yang paling poten dan ekstrak metanol daripada daunnya mencatatkan nilai EC_50_ sebanyak 13.041 ± 0.82 μg/mL. Walau bagaimanapun, ekstrak akueus daun *T. scandens* (TSLAE) telah dipilih bagi membolehkan ia digunakan untuk produk herba tradisional. TSLAE menunjukkan nilai EC_50_ sebanyak 13.76 ± 4.50 μg/mL dalam cerakin DPPH dan tidak toksik terhadap sel normal dengan nilai IC_50_ > 100 μg/mL. Metabolit sekunder dengan potensi antioksidan telah berjaya dikenal pasti menggunakan LC/MS dan perpustakaan rujukan, terutamanya flavonoid (37.5%), fenolik (8.9%) dan polifenol (8.9%). HTS-DPPH ialah teknik yang teguh dan pantas untuk menyaring bahan antioksida dalam ekstrak tumbuhan. Ekstrak tumbuhan tersebut adalah tidak toksik secara *in vitro* dan kaya dengan metabolit sekunder yang menyumbang kepada bioaktiviti.

**Highlights**

- Establishment of a robust high-throughput screening (HTS) of DPPH assay using 384-well plate.
- *Tetracera scandens* extract has potent antioxidant properties that is non-toxic to normal cells.
- Secondary metabolites which are flavonoids, phenolics and polyphenols were identified in *Tetracera scandens* aqueous extract.

## INTRODUCTION

The discovery of novel pharmaceuticals is a complex and multidimensional process that often commences with the identification of bioactive chemicals that can modulate biological processes. In recent years, high-throughput screening (HTS) has become a crucial method in drug development, enabling researchers to effectively assess extensive libraries of compounds for their therapeutic efficacy. HTS utilises automation and sophisticated technologies to perform dozens, or even millions, of tests concurrently, hence expediting the identification of potential candidates for further research. This method not only optimises the preliminary phases of drug development but also improves the capacity to delineate the pharmacological profiles of these compounds, encompassing their efficacy and safety (Ayon 2023; Szymański *et al.* 2012).

Natural products (NPS), as a category of bioactive molecules, have attracted significant interest due to their extensive diversity and longstanding applications in traditional medicine. Antioxidants from natural sources are particularly noteworthy due to their capacity to mitigate oxidative stress, a major role in numerous chronic diseases, including cancer, cardiovascular diseases and neurological disorders. Antioxidants are essential for neutralising free radicals and averting cellular damage. Traditional methods for evaluating antioxidant activity are often laborious and time-consuming, underscoring the necessity for more effective screening techniques (Muscolo *et al.* 2024).

Notwithstanding the progress in HTS technologies, a considerable research gap persists in the systematic identification of natural compounds with strong antioxidant capabilities, alongside the assessment of their safety profiles. Although HTS helps expedite the identification of antioxidant candidates, it is essential to evaluate their cytotoxicity to ensure the safety of these compounds for subsequent research. This study seeks to fill this gap by creating a reliable HTS assay employing the 2-Diphenyl-1-Picrylhydrazyl (DPPH) technique in 384-well format for the evaluation of natural materials. Subsequent to the discovery of hits based on antioxidant activity, cytotoxicity assay will be performed to evaluate their safety profiles prior to phytochemical profiling. The project aims to provide useful insights into the identification of safe and effective natural antioxidants for possible medicinal uses using a comprehensive methodology.

Plants have long been sources of exogeneous antioxidants (such as dietary) with two-thirds of the global species possessing excellent medicinal value (Akbari *et al.* 2019; Sharifi-Rad *et al.* 2020). The interest in exogenous plant antioxidants was initially sparked by the discovery and subsequent isolation of ascorbic acid from plants (Kasote *et al.* 2015). Since then, plants’ antioxidant ability has been generally recognised as an important causative factor in the growth and progression of several life-threatening diseases, including neurodegenerative (ND) and cardiovascular diseases (Mor *et al.* 2021; Sharifi-Rad *et al.* 2020). The antioxidants are small molecules or complex systems, which can scavenge free radicals and protect the oxidisable molecules by delaying, retarding or inhibiting its auto-oxidation if added in small quantities (Alara *et al.* 2019). The antioxidants significantly slow or prevent oxidation of metabolisable substrates when active at lower concentrations than the optimal levels (Santos-Sánchez *et al.* 2019).

HTS is commonly employed in pharmaceutical and biotechnology companies to identify compounds (called hits) with pharmacological or biological activity. HTS has become an integral feature of pharmaceutical research by recognising small organic molecules as potential therapeutics or as samples for a better understanding of biological processes. Wide screening tasks tend to include massive sets of compounds that can deplete resources for time, effort and reagent (Chai *et al.* 2015). Some HTS platforms use cell-based assays to assess processes, such as cell growth/death, binding of receptors or protein expression while others are free of cells that define biological activity. Both formats use several detection technologies, including fluorescence and light readings (Borrel *et al.* 2020).

Luminescence, fluorescence and absorbance are subject to various chemicals, including those that modulate signal strength without any biological intervention. Free radical scavenging measures such as 2,2′-azino-bis (3-ethylbenzothiazoline-6-sulfonic acid) (ABTS) and 2,2diphenyl-1-picrylhydrazyl (DPPH) assays are used to measure antioxidant activity due to their simplicity and capability for high-performance screening. These ABTS and DPPH tests provide details on the mechanisms of antioxidant activity (e.g., transfer of electrons and hydrogen atoms) (Ghani *et al.* 2019). The recently developed HTS assay for antioxidant activity detection is mostly based on the correlation between mass intensity and activity profiles of sequential fractions obtained from liquid chromatography, Fourier transforms (FT) or mass spectrometry (MS) machinery. However, these HTS assays are time-consuming, costly and cater for a limited samples (Park *et al.* 2017). Hence, our team has intensified efforts to upgrade the most popular chemical assay, which is the DPPH assay (de Torre *et al.* 2019), from a conventional 96-well to 384-well by using the HTS platform integrated with our natural product (NP) repository. To date, no research has been carried out to develop the DPPH assay in high-throughput 384-well format.

Malaysia is a tropical country with enormous biodiversity. This study is part of a national collaborative campaign to capture the greatest value from the nation’s biodiversity by uncovering their potential in nutraceuticals and pharmaceuticals developments for therapeutic indications of high-burden diseases in Malaysia. Age-related disorders, including non-communicable diseases (NCDs), are high-burden diseases in Malaysia, accounting for over 72% of total deaths. Older adults, particularly those aged 67.4%, contribute significantly, with ischaemic heart disease and cerebrovascular disease being major causes of premature mortality (Khaw *et al.* 2023; Chan *et al.* 2022). As addressing these disorders is crucial for Malaysia’s health strategy, we have embarked on an HTS programme to search for potential NP agents in combating age-related disorders from our automated repository storage system known as MyNature50000 (http://www.mynature50k.com/) by identifying their antioxidant activity before further evaluating the natural product candidates for cytotoxicity and chemical profiling. Antioxidants are essential in alleviating oxidative stress, associated with the advancement of age-related ailments, including cardiovascular diseases, diabetes and neurological disorders. Ageing results in an imbalance between reactive oxygen species (ROS) and the body’s antioxidant defences, leading to the accumulation of oxidative damage that might worsen various diseases. Consequently, the consumption of antioxidants has been linked to enhanced health outcomes and a diminished risk of age-related ailments, underscoring its potential in fostering good ageing and longevity (Rusu *et al.* 2022; Tan *et al.* 2018). The MyNature50000 repository system stores isolated compounds, fractions, partitions and crude extracts from at least 500 local plants, which are mainly leaves collected from rainforests in Peninsula Malaysia.

The existing literature reveals a research gap concerning the absence of a systematic methodology for creating HTS assays aimed at identifying natural compounds with antioxidant characteristics, specifically employing the DPPH method. This study seeks to develop a reliable HTS method to effectively evaluate natural products for notable antioxidant properties, while concurrently assessing their safety through cytotoxicity analysis and phytochemical characterisation.

## MATERIALS AND METHOD

### Chemicals, Cell Line and Culture Medium

2,2-diphenyl-1-picrylhydrazyl, DPPH (D9132), resazurin sodium salt (R7017) and gallic acid (G7384) were all purchased from Sigma-Aldrich®, US. Ascorbic acid (AB0021) was purchased from Bio Basic Inc., Canada. Human astrocytes (T0281), astrocytes PriGrow IV Medium (TM004) and foetal bovine serum (TM999) were all acquired from ABM Inc., Canada.

### Preparation of Methanol Extracts

The leaf samples were washed carefully with water to remove dirt. Then, 300 g of the leaves were dried in an oven at 60°C until the moisture content was less than 10% (via moisture meter). Approximately 200 g of dried leaves were ground into powder using a grinder and soaked with 600 mL of methanol using the maceration method for three days. The suspension was filtered through filter paper and the filtrates were collected (the extracts). These maceration and filter steps were repeated thrice. The methanol extracts were then dried at 40°C under reduced pressure in a rotary evaporator. Thereafter, 10 mg of each extract was dissolved into 1 mL of DMSO to obtain a final concentration of 10 mg/mL as stock solution and stored at −20°C. For the assay, 363 plant extracts, each extract was prepared in two 96-well plates at a concentration of 100 μg/mL (as technical replicates) to be tested in 384-well assay plates. In this study, 363 methanolic extracts of plant leaf samples were screened for their potential radical scavenging activity of DPPH chemicals by using the HTS system.

### Development and HTS Metrics of Antioxidant DPPH Assay

#### Development of HTS-DPPH assay in 384-well

- **Spectra determination of absorption reading:** The radical scavenging activity of plant extracts and standard antioxidants was measured by direct hydrogen donation to the DPPH radical following the method reported previously (Proestos *et al.* 2013; Yang *et al.* 2011) with minor modifications and adaptation to fit HTS experimental requirements. By using an automated liquid handler (NIMBUS MICROLAB^(R)^, Hamilton) that is attached to a laptop, the aspiration, dispensing and mixing steps into the 384-well plate were performed according to the protocol programming series developed based on the following assay. Sodium acetate buffer pH 6.0 was prepared by adding 0.1 M glacial acetic acid with 0.1 M sodium acetate tri-hydrate for dilution in a total volume of 12.5 μL. The initial concentration of the standard (gallic acid), 100 μg/mL, was prepared with sodium acetate buffer and 12.5 μL was loaded into the 384-well assay plate. Next, 25 μL of the DPPH solution (0.5 mM) prepared in ethanol was added into the wells resulting total volume of 50 μL/well and was incubated for 30 min. Finally, spectral scanning of the mixture was carried out using the microplate reader (BioTek®, US).
- **Optimisation of DPPH concentration:** The protocol described in Section (a) was repeated with various concentrations of DPPH starting from 0.05 mM, 0.1 mM, 0.2 mM and 0.4 mM and the absorbance was determined via the spectral scanning taken for each concentration.
- **Linearity of standard concentration (A_530_):** The protocol described in Section (a) was repeated with predetermined absorbance spectral and DPPH concentration, by using a serial concentration of the standard (gallic acid); 100 μg/mL, 50 μg/mL and 25 μg/mL, which was prepared with sodium acetate buffer. The standard was then loaded into the 384-well assay plate. Next, the DPPH reagent was added with its predetermined concentration before recording the absorbance. Apart from the linearity analysis based on the graph pattern, the concentration of the standard with an absorbance reading of < 0.5 was selected for further analysis.

#### Sensitivity of the HTS assay

The HTS assay protocols were conducted in an optimum condition while the sensitivity and quality were determined by calculating HTS assay metrics via three equations (Equations 1, 2 and 3), comprising the values of Z factors (Z’), signal to background ratio (S/B) and percentage of coefficient of variation (% CV) (Ollinger *et al.* 2019).

Z factor formula:


(1) $$Z^{\prime }=1\left(\frac{3{SD}_{max}+3{SD}_{min}}{{Mean}_{max}-{Mean}_{min}}\right)$$

Signal-to-background (S/B) ratio formula:


(2) $$S/B=\frac{{Mean}_{max}}{{Mean}_{min}}$$

Percentage of coefficient of variation (%C/V) formula:


(3) $$\%C/V=\frac{SD}{Mean}$$

where, SD = standard deviation; max and min in the subscripts are calculated from values taken from A_530_ readings of background (max) and samples (min) upon completion of the assay’s protocol. In this case, samples are the readings obtained from the standard (gallic acid).

### Primary and Secondary Screening (via DPPH Assay) for Identification of Positive Hits

A 384-well plate was used (Kawahara *et al.* 2012) for the primary screening of 363 plant extracts that were prepared in duplicates. In the primary screening, the concentration of a tested substance (extracts and standards) used are 50 μg/mL. The selection of plant extracts with potent inhibitory effect was based on the colourimetric observation, a ‘yes’ or ‘no’ score in colour changing upon incubation at the end of the assay. Antioxidative agents in plant extracts reduced the DPPH colour from purple to pale yellow, indicating a ‘yes’ score. The ‘yes’ score samples were further confirmed by reading the adsorption at A530nm via a multimode plate reader (BioTek®, US). The readings for ‘yes’ score samples were below 0.5, as the blank (without samples) reading was around 1.0. The sample was classified as a ‘no’ score when the pale purple colour remained unchanged.

During the secondary screening for confirmation of the scores, the ‘yes’ score samples were retested using the same method. This process started by loading the assay buffer into the assay plate until the mixture was left to stand for 30 min in the dark. At this stage, the extracts were tested in a dose-response manner in duplicates at four different concentrations, ranging from 1.85 μg/mL to 50 μg/mL, to obtain the EC_50_ values. The EC_50_ values of gallic acid, ascorbic acid and extracts were estimated in a dose-response manner, and the radical scavenging rate at each dose point was calculated. The scavenging rate was calculated using the following formula:


(4) $$Scavenging\;rate=\left(1-\frac{A_{1}-A_{2}}{A_{0}}\right)\times 100\%$$

where A_0_ is the absorbance of the control without sample, A_1_ is the absorbance in the presence of the sample (signal) and A_2_ is the absorbance of the sample without DPPH radical (background) (Yang *et al.* 2011). A_0_ and A_2_ controls are needed to eliminate false-positive results. The formula uses A_0_ as a reference to normalise results against a baseline, focusing on DPPH absorbance, ensuring accurate capture of changes. It calculates a relative scavenging percentage based on the sample’s action, considering the actual signal change relative to DPPH. This method avoids false positives from unrelated variations in sample absorbance. The effective concentration at which 50% of DPPH radicals were scavenged, expressed as the EC_50_ value (Chen *et al.* 2013; Molyneux 2004) which was obtained by plotting the percentage of scavenging rate (%) against the concentration using GraphPad Prism software (version 5.0). Assays were performed in duplicates in three independent experiments.

### Aqueous Extract Preparation of ***T. scandens*** Leaves

The leaves of *T. scandens* Linn. Merr. were collected from a village in Kampung Kulim, Kedah, Malaysia in 2017 and the authentication was performed by a botanist in Universiti Sains Malaysia (USM). A voucher specimen (PID 070412-01) was deposited into the Herbariums in IPharm and the School of Biological Sciences, USM. The leave extracts were prepared as stated in the previous section (extract preparation for 363 plants), starting from washing of the leaves until the filtrate was collected using distilled water as a substitution for methanol. The aqueous extracts were then freeze-dried. Next, 10 mg of extract was dissolved into 1 mL of DMSO to obtain a final concentration of 10 mg/mL as stock solution and stored at −20°C for further assay.

### Astrocyte Culture Condition

Astrocytes were cultivated as reported previously (Rosenberg *et al.* 2018) in an astrocyte complete medium, which consists of PriGrow IV Medium (TM004) and 10% of foetal bovine serum (TM999) obtained from ABM Inc., Canada. Astrocytes were grown in an extracellular matrix (ECM) coated T-25 vented cap flasks with a humidified atmosphere of 5% CO_2_ at 37°C. The cells were sub-cultured at three days intervals with an initial concentration of 2 × 10^5^cells/mL.

### Cytotoxicity Evaluation

Cytotoxicity assay was conducted as reported by prior researchers (Zahari *et al.* 2014). Briefly, each well of a 96-well microtiter plate contained 100 mL of 5 × 10^4^ cells/mL astrocytes in a complete culture medium. The medium was replaced after 24 h with a freshly prepared complete medium prior to incubation in the presence of identified extracts with antioxidant activities at concentrations in the range of 0.137 and 100 μg/mL for 72 h. The concentration of DMSO in the wells with the highest sample concentration did not exceed 0.5%. The background fluorescence of the sample containing medium with cells was determined for each dilution, whereas the wells without extracts (compounds or agents) served as negative controls. In the context of cytotoxicity assays, using background fluorescence from samples containing medium with cells as a negative control serves several important purposes. It provides a baseline measurement reflecting the inherent properties of cells and the medium, allowing assessment of fluorescence due to experimental conditions versus that due to the presence of cells. Establishing this background as a negative control accounts for non-specific fluorescence, ensuring observed effects are genuinely due to cytotoxicity rather than artifacts from the assay setup. Wells without extracts serve as a comparison point for assessing treatment effects, while using both background and negative controls helps normalise data across different experimental conditions, ultimately enhancing data reliability and supporting better conclusions about the cytotoxic potential of tested substances. Gallic acid and ascorbic acid (vitamin C) which are known as potent antioxidants were used as positive controls in the cytotoxicity assay. The plates were incubated for 70 h at 37°C per 5% CO_2_. Thereafter, 10 μL of resazurin was added into each well and the plates were incubated for another two hours until the colour of resazurin changed from blue to pink. The plates were read with a fluorescence plate reader (BioTek®) using excitation and emission wavelengths of 530 nm and 590 nm, respectively. GraphPad Prism software (version 5.0) was employed to determine the inhibitory concentration, which reduced the cell viability to as much as 50%, also known as the IC_50._ The wells were screened in triplicate over three independent experiments.

### Statistical Analysis

All data were expressed as mean ± standard error of the mean (SEM). The dose-response graph was constructed using GraphPad Prism software (version 5.0). The means between independent experiments were compared using one-way ANOVA.

### Mass Spectrometric Analysis

Compound separation was carried out using an Agilent Technologies 1290 Infinity liquid chromatography system equipped with a quaternary pump (G4204A), an autosampler, a HiP Sampler (G4226A) and a column warmer (G1316C). Agilent MassHunter Workstation software version B.06 was used to control the instrument and analyse the results. At 30°C, a Zorbax Eclipse Plus C18 analytical column (Rapid Resolution HD, 2.1 × 50 mm, 1.8 m) was employed for chromatographic separation. The mobile phase was composed of methanol (Solvent B) and water (0.1 mL formic acid/100 mL water), while the flow rate was maintained at 0.2 mL/min. The gradient elution began with a 5% B to 95% B gradient for 0 to 10 min, followed by 95% B for 10 to 11 min, 95% B for 11 to 12 min, and 5% B for 12 to 15 min. The injection volume was 2 μL. Mass spectrometric analysis was performed (G6540B) on a 6540UHD Accurate Mass Q-TOF LC/MS. The mass spectra were acquired in the ionisation mode across the mass range m/z 100–1700. Additional mass spectrometer conditions included the following conditions: 35 psi nebulising gas pressure; 8 L/min drying gas flow; and 300°C drying gas temperature. The chemicals were analysed using the unique negative ionisation modes (m/z [M-H]).

## RESULTS

### Development and HTS Metrics of Antioxidant DPPH Assay

#### (a) Determination of spectra, DPPH concentration and assay linearity

**Figure 1:**
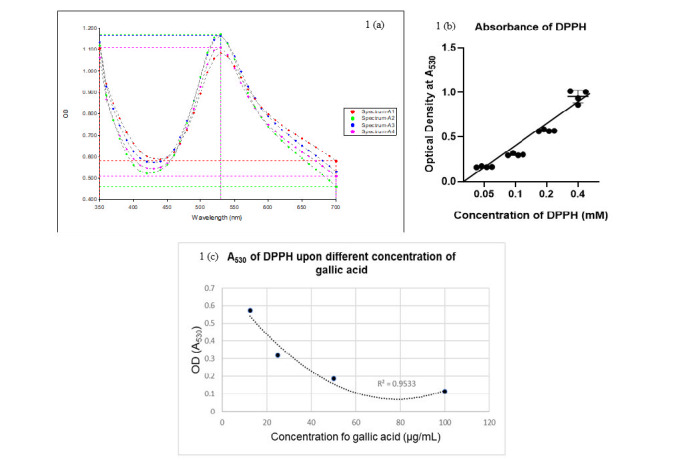
(a) A spectral scanning resulted at 530 nm; (b) A_530_ for different concentrations of DPPH; and (c) Regression analysis of the DPPH assay. *Notes*: The absorbance spectrum of the substrate (DPPH) was measured in quadruplicates using a 384well assay format, ensuring statistical reliability and quality control. The highest value approaching 1.0 was at 530 nm, aligning with previous studies. The assay showed a non-linear relationship between gallic acid concentrations and optical density, suggesting factors like saturation effects or interactions between antioxidants and DPPH radicals at higher concentrations. This aligns with previous studies.

The optimisation of spectral scanning or range of optical density (OD), substrate concentration and assay linearity are the basis of HTS assay development (Jones *et al.* 2016; Lim *et al.* 2016). Fig. 1(a) shows the absorbance spectrum of the substrate (DPPH) starting from 350 nm to 700 nm in quadruplicates, and the highest value approaching 1.0 was at 530 nm. Fig. 1(b) portrays an A_530_ for a serial range of DPPH concentration. At concentrations ranging from 0.2 mM to 0.4 mM, the A_530_ values were approaching 1.0 and directly proportional to the DPPH concentration. Meanwhile, Fig. 1(c) presents that the developed assay has a non-linear regression with a quadratic best-fit curve, yielding a higher R^2^ value compared to the linear pattern, which is 0.9533.

#### (b) QA metrics of HTS-DPPH assay

The purple-coloured DPPH radicals in the solution turned yellow upon interacting with the antioxidative extract. This activity was quantified by measuring the absorbance at 530 nm (A_530_). Assay validity was assessed based on the quality assurance (QA) parameters of the high-throughput screening (HTS) assay, which include Z’ factor, signal-to-background (S/B) ratio and coefficient of variation (% CV). The A_530_ values were utilised in the calculations for these metrics, yielding results of 0.72 for Z’, 3.54 for S/B ratio and 4.25 for % CV during primary screening. In secondary screening conducted in a dose-response manner, the values were 0.63 for Z’, 9.02 for S/B ratio and 6.49 for % CV. The summarised values in Table 1 indicate that both assays were robust and met the criteria for an excellent HTS assay (Sui & Wu 2007). The QA metrics were obtained from the development of the DPPH assay by adopting the HTS system to screen for positive hits from our plant extract repository, MyNature50000.

##### Primary screening and EC_50_ values in the secondary screening of the MyNature50000 repository

**Figure 2:**
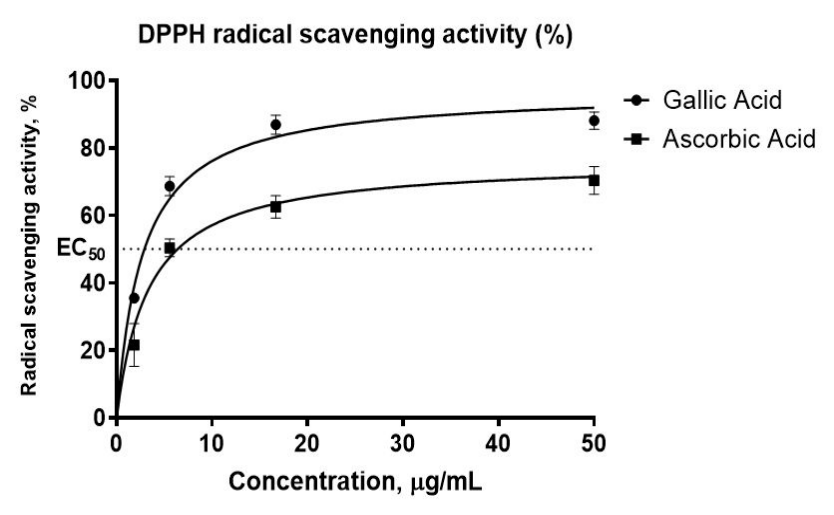
DPPH radical scavenging effect of ascorbic acid, gallic acid using a 384-well plate and the HTS programme in dose-response within the range of 1.85 μg/mL–50 μg/mL. *Notes.* The results were analysed as the effective concentration at which 50% of the DPPH radicals were scavenged, EC_50_ in μg/mL. Data presented as means ± SEM from duplicates over three other independent experiments, with *P* < 0.05 which was not significantly different between all independent experiments. EC 50 was calculated to be 6.48 ± 0.75 μg/mL for ascorbic acid and 3.48 ± 0.22 μg/mL for gallic acid.

In the primary screening, the colour change for the two standard antioxidants (ascorbic acid and gallic acid) took place at only one concentration, which is 50 μg/mL. Fig. S2 in Appendix B illustrates an example of the results obtained by observing the colour change of a 384-well plate during the primary screening. Thus, further evaluation of EC_50_ in the secondary screening for dose-response curve started at 50 μg/mL and below. Antioxidant screening programmes of MyNature50000 extracts via HTS-DPPH assay development began with obtaining the EC_50_ values for standards. The standards for gallic acid (GA) and ascorbic acid (AA) revealed EC_50_ values of 3.48 ± 0.22 μM and 6.48 ± 0.75 μM, respectively (Fig. 2).

**Figure 3:**
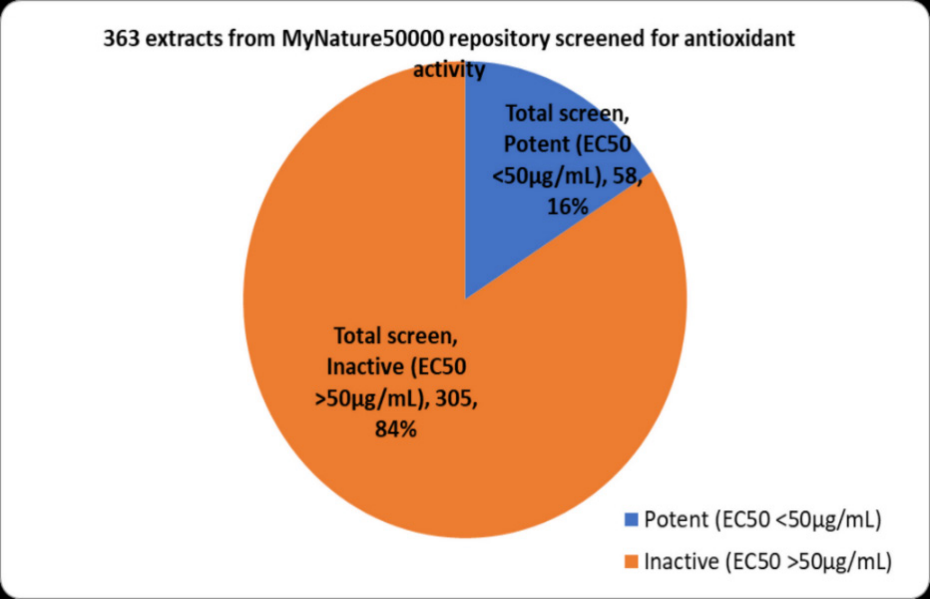
Chart showing the percentage of a ‘yes’ score (16%) in the primary screening among the 363 total extracts screened of which only 80 plant extracts owned herbarium. *Notes.* Based on primary screening of 363 extracts and a further assessment of 80 extracts accessible during the research, 16%, equating to 58 plants, were potent antioxidant with EC_50_ values < 50 μg/mL. Conversely, 84% were deemed ineffective antioxidants in the screening program, as the EC_50_ values exceeded 50 μg/mL.

Based on the primary screening of 363 extracts and secondary screening of 80 extracts that were available during the project, 116% (58) of the plants were considered potent with EC_50_ values of less than 50 μg/mL (Fig. 3). On the other hand, 84% were not considered as potent antioxidants in the screening programmes, as the EC_50_ values were greater than 50 μg/mL. However, not all the 363 plants were verified by a certified botanist based on their scientific names identification and herbarium confirmation. In other words, some plants are still in the process of identification, as the earlier process of identification prior herbarium was based on traditional methods. Conventional techniques for plant species identification encompass expert assessment, recognition, comparative analysis, dichotomous keys, morphological examination and field guides. Experts utilise information from monographs, revisions, or floras, whereas identification depends on acquaintance with particular plant groups. Comparison entails evaluating unknown specimens against known ones, albeit it can be time consuming and labour-intensive. Identification keys assist users in making selections based on discernible plant traits, whereas morphological analysis entails meticulous examination of plant structures.

According to the screening result of 363 plant extracts depicted in Fig. 3, 16% of them fall under this category. Overall, 12 extracts showed the most potent antioxidant activities belonging to *T. scandens*, followed by *Adinandra dumosa* < *Gluta lanceolata* < *Cinnamomum porectum* < *Dillenia reticulate* < *Eleiodoxa conferta* < *Pternandra coerulescens* < *Mangifera graffiti < Calophyllumcalaba* var *bracteatum* < *Barringtonia macrostachya* < *Streblus elongates* < *Nauclea orientalis* as listed in Table 2 alongside with other 80 identified plant species. To proceed with the next determination, the most potent antioxidant activity in the list was chosen and its aqueous extract for subjected to further analysis, that is *T. scandens* with EC _50_ value = 13.041 ± 0.82 μg/mL. TSLAE has similar patterns to dose-response curves of EC_50_ standards and the calculated value was 13.76 ± 4.50 μg/mL. Appendix Fig. 3 presents an example of the herbarium; *T. scandens*, identified by a certified botanist as mentioned in the methodology.

### Cytotoxicity Evaluation of TSLAE

**Figure 4:**
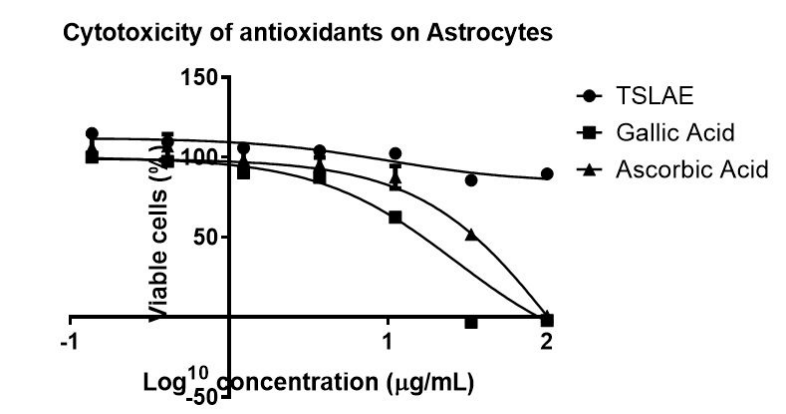
Cytotoxicity assay was validated with IC_50_ values of two standard antioxidants which are gallic acid = 15.31 ± 0.37 μg/mL and ascorbic acid = 36.16 ± 4.20 μg/mL. *Notes.* Astrocytes were cultured for 72 h in the presence of gallic acid, ascorbic acid and TSLAE at concentrations from 0.137 μg/mL–100 μg/mL. The results were analysed as a percentage of viable astrocytes in relation to the untreated ones. Data presented as means ± SEM from triplicates over three other independent experiments, with *P* > 0.05 which was not significantly different between all independent experiments. There were more than 80% of viable cells even at the highest concentration of TSLAE used (100 μg/mL).

The cytotoxicity of TSLAE on astrocytes was determined using the AlamarBlue® assay. The dose-response curve was obtained by plotting the percentage of viable cells (%) vs. log concentration of the extract. The assay was validated using two standard antioxidants (Fig. 4), which are gallic acid and ascorbic acid, with IC_50_ values of 15.31 ± 0.37 μg/mL and 36.16 ± 4.20 μg/mL, respectively (Filipiak *et al.* 2014). The IC_50_ value of TSLAE was > 100 μg/mL.

### Phytochemical Analysis of TSLAE

**Figure 5:**
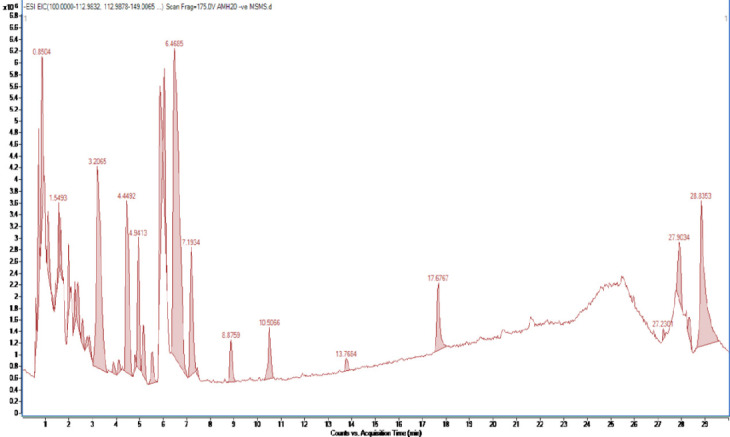
LC/MS chromatogram of TSLAE. *Notes.* The LC/MS analysis identified 54 main chemicals that coincide with Table 3 and Table 4, that showed a total percentage of flavonoids (37.5%), polyphenols (8.9%), and phenolic compounds (8.9%) in the TSLAE. These comprise well-known strong antioxidants: gallic acid (mass = 170.02, Rt = 0.9778 min), quercetin derivatives (mass = 464.09, Rt = 3.3141 min), and kaempferol (mass = 286.05, Rt = 4.4529 min).

LC/MS studies were carried out on TSLAE with chromatogram as shown in Fig. 5. The LC/MS was interpreted for the identification of 54 predominant compounds as presented in Table 3. The majority of these compounds were reported to exist in various *T. scandens* extracts in the previous studies (Table 4). Meanwhile, the present study implied TSLAE and managed to identify a total percentage of flavonoids (37.5%), polyphenols (8.9%), and phenolic compounds (8.9%). These compounds included the commonly known potent antioxidants that are gallic acid (mass = 170.02, Rt = 0.9778 min), quercetin derivatives (mass = 464.09, Rt = 3.3141 min), and kaempferol (mass = 286.05, Rt = 4.4529 min).

## DISCUSSION

In high-throughput screening, the choice of assay format and measurement methodology plays a crucial role in ensuring data reliability and quality. Quadruplicates in a 384-well assay format improve statistical reliability, reduce variability and enhance quality control. They provide four independent measurements, identify outliers and facilitate comparison across conditions, making it a standard practice in high-throughput screening. This outcome aligns with procedures indicating that measurements should be conducted at 530 nm (Abourashed 2005), despite Abourashed (2005) employing a 96-well plate while our study utilised a 384-well plate. The A_530_ values that were approaching 1.0 and directly proportional to the DPPH concentration aligned with the reports from the previous studies (Kedare & Singh 2011). The non-linear regression best-fit curve that showed in the relationship between gallic acid concentrations (independent variable) and optical density (dependent variable) is not strictly linear. This non-linear relationship can occur due to various factors, such as saturation effects or interactions between the antioxidant and DPPH radicals at higher concentrations. The incubation time of the DPPH assay was kept at 30 min as all three parameters in Fig.1 were taken at 30 min of assay incubation.

The values obtained in both first and second screening assays fulfilled the criteria for excellent HTS, with Z-prime (Z’) values within the range of 0.5 < Z’ < 1, S/B approaching 10 and % CV values less than 10% (Sui & Wu 2007; Sykes & Avery 2009). The DPPH colourimetric assay is a widely used method for free radical scavenging due to its rapid, cost-effective nature and minimal reagent requirements (Alam *et al.* 2013). Traditionally conducted in 96-well plates, this assay has evolved into HTS using 384-well plates and robotic automation linked to compound libraries (Paricharak *et al.* 2018). HTS facilitates extensive and efficient screening processes, requiring robust data processing tools for quality assessment (QA) (Goktug *et al.* 2013). The findings by Sui and Wu (2007) support the integration of metrics like the Z factor and power analysis into HTS assays, enhancing their reliability and contributing to effective drug discovery. As HTS has become essential in drug development, it enables the evaluation of large compound collections while ensuring rigorous validation to confirm assay robustness and reliability (Chai *et al.* 2015). This study aims to simplify HTS for antioxidant activity evaluation through automated DPPH methods based on sequential injection analysis (SIA) and dot blot assays that rely on colour intensities. Gallic acid and ascorbic acid are chosen as standard reference compounds in antioxidant assays due to their established efficacy, ability to provide comparative data across studies, and their role in ensuring methodological consistency in evaluating antioxidant activities (Kedare & Singh 2011; Sarian *et al.* 2017). The rationale behind conducting primary and secondary screening assays lies in their ability to systematically identify and confirm active compounds. Primary screening serves as an initial filter to detect potential hits with significant antioxidant activity, while secondary screening allows for a more detailed dose-response evaluation of these hits, ensuring that only the most promising candidates are selected for further investigation.

The values of gallic acid and ascorbic acid obtained were similar to the values reported previously (Lu *et al.* 2014; Chanda & Nagani 2010), validating the developed assay. Ascorbic acid also has the most established data in all antioxidant assay methods, such as in determining the kinetics, stoichiometric and standard correlation or regression graph analyses (Amorati & Valgimigli 2018). Meanwhile, gallic acid is frequently used in assays related to total phenolic content and serves as one of the assay standards. This might be attributed to the fact that gallic acid is amongst the strongest antioxidant compounds in edible plants, one of the most abundant phenolic acids in plants, and has diverse scientific reports on biological and pharmacological activities (Kahkeshani *et al.* 2019). A clear negative control (background) of the assay should be described and assigned to avoid false-positive results of any assay (Chai *et al.* 2015). Therefore, in this DPPH assay, the background is the reading of extract with buffer system and without DPPH radical.

Using 50 μg/mL as a cutoff for EC_50_ concentration in antioxidant assays provides a standardised, effective, and practical approach to evaluating the antioxidant activity of various compounds. It facilitates meaningful comparisons and ensures that results are relevant to biological systems while maintaining feasibility in experimental design (Milanezi *et al.* 2019). The classification of potency is based on the previous research (Phongpaichit *et al.* 2007), which studied antioxidant from fungal extracts using DPPH assay and classified the results as IC_50_ > 250 μg/mL as inactive; > 100–250 μg/mL as weakly active; > 50–100 μg/ mL as moderately active; 10–50 μg/mL as strongly active; and < 10 μg/mL as very strongly active. Thus, this article focused on 80 plants that have been identified by a botanist based on their genus, species and variant names. In addition, these plants have been deposited into the herbarium, whereas the remaining plants were reported in the database using their common and local names at the repository (http://www.mynature50k.com/).

Aqueous extracts are often chosen in herbal product development due to their safety, acceptability, targeted phytochemical profile, antioxidant activity, cost-effectiveness and regulatory considerations. They are safer, more acceptable and have lower toxicity profiles, making them a practical choice for large-scale production and testing. Aqueous extracts and methanol extracts may contain similar secondary metabolites due to their polarity and antioxidant activity. Both types of extracts have similar total phenolic content and antioxidant activity, possibly due to the presence of similar compounds found in methanol extracts (Goktug *et al.* 2013; Sharma & Vig 2013).

*Tetracera scandens* has been reported to possess anti-diabetic (Umar *et al.* 2010), anti-HIV (Tlili *et al.* 2013) and antibacterial activities (Muliyah *et al.* 2018). The ethanolic extract of *T. scandens* was also reported to exhibit antioxidant properties in acute liver injury in rats (Thanh *et al.* 2015), whereas the methanolic extract was found to exhibit antioxidant activity via the DPPH scavenging assay (Uddin Mazumdar *et al.* 2017). Additionally, its methanol-water extract showed strong xanthine oxidase (XO) inhibitory activity that may be useful for the treatment of hyperuricemia and gout (Nguyen *et al.* 2004). The extract also displayed potent XO inhibitory activity with an IC_50_ value of 15.6 μg/mL compared to another extraction method, methanol and water with IC_50_ values of 33.3 μg/mL and 25.5 μg/mL, respectively. The potent antioxidant activity of TSLAE may be due to the flavonoids and terpenoids in the plant, such as kaempferol, quercetin, isoscutellarein, hypolaetin, astragalin and stigmasterol derivatives that was also detected in methanol extract of the plant species (Ahmed *et al.* 2014). Flavonoids are known for scavenging free radicals derived from H_2_O_2_ (Treml & Šmejkal 2016). Several flavonoids were also identified in the genus of *Tetracera*, specifically from *T. indica* and *T. scandens* methanol extracts which showed antioxidant activities such as wogonin, methyl-ether, acetate, techtochrysin, 8-Hydroxy-7-methoxyflavone, chrysin, norwogonin, acetate (norwogonin), isoscutellarein, hypolaetin, kaempferol, quercetin, (+)-catechin and (−)-epicatechin (Sarian *et al.* 2017).

*T. scandens* is a flowering plant that belongs to the family of *Dilleniaceae*, also known as ‘akar mempelas’, ‘mempelas kasar’, ‘palas’ and ‘mampan’ and other synonyms, such as *Delima sarmentosa* L., *Tragia scandens* L., *Tetracera monocarpa* and *T. hebecarpa* (Umar *et al.* 2010). They are widely grown in Malaysia, China, Myanmar, Indonesia, the Philippines, Vietnam and Thailand. Different parts of *T. scandens* have been ethnobotanically effective for the treatment of metabolic ailments, including rheumatism, hypertension, blood pressure, inflammatory diseases and diabetes (Lima *et al.* 2014). These therapeutic and pharmacological applications are largely due to the presence of natural antioxidant constituents in the *T. scandens* cellulosic matrix.

Each research on herbal medicine outcome targets different needs, active ingredients or formulations. The herbal materials used in traditional herbal preparation are often extracted with water to make an aqueous extract or decoction (Wang *et al.* 2019).

TSLAE was found as non-toxic on astrocytes. To date, there is no cytotoxic data of *T. scandens* aqueous extract. Nevertheless, there is one report for the ethanol (70%) extract, with IC_50_ > 40 μg/mL in human T-cells (Kwon *et al.* 2012). Additionally, ethanol extract of *T. scandens* was found as non-toxic to rats (Umar *et al.* 2010) and its methanol extract demonstrated a toxicity value of > 400 mg/kg body weight of mice (Uddin Mazumdar *et al.* 2017). Astrocytes, one of the largest glial cell populations in the central nervous system (CNS), perform a myriad of functions to maintain homeostasis and support neuronal function (Matias *et al.* 2019). Astrocyte was chosen in the current and future studies for oxidative stress and brain ageing related to NDs as it was found to be suitable for stress-induced senescence (SIS) ageing in *in vitro* model, which we are currently working on. Furthermore, the population of senescent astrocytes increases in the human brain during ageing, especially in Alzheimer’s disease (Bhat *et al.* 2012).

The cumulative effects of these secondary metabolites are responsible for the inherent therapeutic and pharmacological effects of *T. scandens*, especially in radical scavenging activity of the overproduction and accumulation of free radicals. The secondary metabolites, such as flavonoids and phenolic compounds, can neutralize free radicals (Lobo *et al.* 2010), interrupt oxidative chain reactions, modulate gene expression (Kurutas 2016), chelate transition metal ions (Zduńska *et al.* 2018), prevent lipid peroxidation (Pehlivan 2017) and regulate antioxidant enzymes (Goktug *et al.* 2013).

Gallic acid, a trihydroxybenzoic acid acts by providing efficient protection against oxidative damage caused by reactive species often encountered in biological systems, including hydroxyl (HO^•^), superoxide (O_2_^•^−) and peroxyl (ROO^•^), as well as the non-radicals, such as hydrogen peroxide (H_2_O_2_) and hypochlorous acid (HOCl) (Badhani *et al.* 2015). These features of gallic acid are responsible for the apoptosis of cancer cells and protection against cardiovascular, neurodegenerative and metabolic diseases (Gao *et al.* 2019) that the gallic acid compound was also detected in TSLAE showed in Table 3. Meanwhile, quercetin is a plant-based flavonoid whose dietary antioxidants have been reported with promising therapeutic potentials against Alzheimer’s disease (Khan *et al.* 2019), breast cancer (Ezzati *et al.* 2020) and glioblastoma multiforme (Tavana *et al.* 2020). Moreover, kaempferol is a flavonoid with antioxidant and anticancer activities as affirmed in prior studies (Imran *et al.* 2019; Ren *et al.* 2019).

## CONCLUSIONS

The present study applied the HTS system with a 384-well plate to conduct the DPPH antioxidant assay. Although the assay is well known and already established in 96-well plates, this time we used the automated liquid handler that was employed to perform the primary and secondary screening programmes with aspiration and dispensing automation in a 384-well microplate format. The most potent plant with the highest antioxidant capacity from the screening results was thereafter subjected to a cytotoxicity study. The HTS assay was optimised using Z-prime (Z’) values of 0.72 and 0.633, signal to background (S/B) ratios of 3.54 and 9.02, and coefficient of variation percentages (%C/V) of 4.25 and 6.49 for the primary and secondary screenings, respectively, thereby indicating an excellent HTS assay. A total of 58 extracts (16%) demonstrated potent antioxidant activities out of 363 extracts screened via the first screening. Eighty (80) out of the 363 plant extracts were identified by the botanist and only 12 of them demonstrated EC_50_ in the HTS-DPPH during the second screening. The present results succinctly revealed *T. scandens* extracts as having the highest antioxidant EC_50_ value of 13.041 ± 0.82 μg/mL and 13.76 ± 4.50 μg/mL for its methanol extract and aqueous extract, respectively. Moreover, the 54 identified secondary metabolites from the extracts were responsible for the *in vitro* antioxidant activities estimated. However, the *T. scandens* extract reflected a non-toxic activity towards normal human astrocytes with an IC_50_ value of >100 μg/mL as determined via AlamarBlue® assay. Overall, these findings may guide researchers with big data or a repository to finalise a few or even a single drug or pharmaceutical candidate via HTS assay and hits-to-lead path. Nevertheless, the present study used plant extract to target traditional medicine (TM) outcomes. The results of those assays could be used as the basis for future studies related to the potential of *T. scandens* in managing metabolic and age-associated disorders, as well as NDs.

## Figures and Tables

**Table 1:** QA metrics of both 1st and 2nd screening assays for DPPH radical scavenging activity via HTS system. Both screening assays were robust and reproducible that fell within the criteria for excellent HTS according to Sui and Wu (2007).

Metrics of HTS assay development^a^	Value (1st screening)	Value (2nd screening)
Z-prime (Z’)	0.72	0.63
Signal to background (S/B) ratio	3.54	9.02
Coefficient of variation (%CV)	4.25	6.49

*Notes.*

a Results normalisation which captured the absorption readings of signal, blank and control are shown in Appendix A.

**Table 2:** Screening results of HTS-DPPH assay from 80 entries (extracts from the identified plant species) of MyNature50000 repository. Each entry has been verified for the codes (appeared in IPNAT numbers), botanical and local names. The first screening results were presented in positive (‘yes’) or negative (‘no’) scores for DPPH reagent colour changes, as well as optical density (OD) values at A_530_. Positive results for primary screening were subjected to secondary screening for antioxidant activity confirmation through a dose-response for EC_50_ values determination.

No.	IPNAT codes in MyNature50000 repository	Botanical name	Local name	Form of extract (methanol or aqueous extract)	First screening scores (‘Yes’ or ‘ No’ colour change; A_530_ value at 50 μg/mL)	Second screening (EC_50_ μg/mL for DPPH radical scavenging assay)
Gallic Acid (Standard antioxidant 1)				-	Yes; 0.390 ± 0.06	3.48 ± 0.22
Ascorbic Acid (Standard antioxidant 2)				-	Yes; 0.407 ± 0.05	6.48 ± 0.75
1	IPNAT00148a/M01	*Avicennia alba*	Api Api Putih	Methanol	No; 0.668 ± 0.07	> 50
2	IPNAT00290a/M01	*Anisophylla grandis*	Delek	Methanol	No; 0.647 ± 0.27	> 50
3	IPNAT00118a/M01	*Mangifera graffithii*	Rawa	Methanol	Yes; 0.368 ± 0.06	29.059 ± 0.60
4	IPNAT00156a/M01	*Gluta lanceolata*	Rengas	Methanol	Yes; 0.333 ± 0.01	15.946 ± 0.82
5	IPNAT00424a/M01	*Swintonia* sp.	Merpauh	Methanol	No; 0.872 ± 0.03	> 50
6	IPNAT00180a/M01	*Buchanania sessilifolia*	Ketak Udang, Pauh Pipit	Methanol	No; 0.733 ± 0.04	> 50
7	IPNAT00147a/M01	Alstonia angustiloba	Pulai	Methanol	No; 0.522 ± 0.14	> 50
8	IPNAT00109a/M01	*Willughbeia angustifolia*	Akar Getah Gaharu	Methanol	No; 0.625 ± 0.20	> 50
9	IPNAT00166a/M01	*Alstonia macrophylla*	Pulai Penipu Bukit	Methanol	No; 1.164 ± 0.04	> 50
10	IPNAT00146a/M01	*Aglaonema Donna Carmen*	Keladi	Methanol	No; 1.350 ± 0.05	> 50
11	IPNAT00185a/M01	*Pinanga malaiana*	Pinang Hutan	Methanol	No; 1.159 ± 0.08	> 50
12	IPNAT00152a/M01	*Caryota mitis*	Fishtail Palm (Dudar)	Methanol	No; 0.724 ± 0.04	> 50
13	IPNAT00259a/M01	*Eleiodoxa conferta*	Kelubi	Methanol	Yes; 0.255 ± 0.07	25.796 ± 1.32
14	IPNAT00124a/M01	*Thottea defenden*	Hempedu Beruang	Methanol	No; 0.611 ± 0.16	> 50
15	IPNAT00252a/M01	*Dracaena fragrans*	Keladi	Methanol	No; 0.717 ± 0.11	> 50
16	IPNAT00202a/M01	*Pajanelia longifolia*	Beka	Methanol	No; 0.646 ± 0.12	> 50
17	IPNAT00186ab/M01	*Dacryodes* sp.	Kedondong	Methanol	No; 0.656 ± 0.10	> 50
18	IPNAT00127a/M01	*Calophyllum calaba* var.*bracteatum*	Bintangor	Methanol	Yes; 0.405 ± 0.02	30.971 ± 4.39
19	IPNAT00120a/M01	*Atuna penangiana*	Merbatu	Methanol	No; 0.666 ± 0.17	> 50
20	IPNAT00159a/M01	*Garcinia mangostana*	Manggis	Methanol	No; 0.862 ± 0.06	> 50
21	IPNAT00141a/M01	*Garcinia pyrifera*	Manggis Hutan	Methanol	No; 0.740 ± 0.13	> 50
22	IPNAT00065a/M01	*Ctenolophon parvifolius*	Mertas	Methanol	No; 1.142 ± 0.04	> 50
23	IPNAT00137a/M01	*Tetracera indica*	Akar Mempelas	Methanol	No; 0.512 ± 0.11	> 50
24	IPNAT00153a/M01	*Dillenia suffruticosa*	Simpoh Air	Methanol	No; 1.021 ± 0.08	> 50
25	IPNAT00269a/M01	*Dillenia reticulata*	Simpoh Gajah	Methanol	Yes; 0.347 ± 0.01	20.500 ± 0.97
26	IPNAT00348a/M01	*Dipteris conjugata*	Payung Ali	Methanol	Yes; 0.500 ± 0.04	> 50
27	IPNAT00270a/M01	*Shorea* sp.	Meranti	Methanol	No; 0.648 ± 0.24	> 50
28	IPNAT00347a/M01	*Dryobalanops aromatica*	Kapur	Methanol	No; 1.241 ± 0.00	> 50
29	IPNAT00201a/M01	*Mallotus leucodermis*	Balik Angin	Methanol	No; 0.483 ± 0.15	> 50
30	IPNAT00158a/M01	*Hevea brasiliensis*	Getah	Methanol	No; 0.841 ± 0.27	> 50
31	IPNAT00229a/M01	*Macaranga* sp.	Mahang	Methanol	No; 0.685 ± 0.29	> 50
32	IPNAT00155a/M01	*Macaranga tanarius*	Mahang Putih	Methanol	Yes; 0.536 ± 0.09	> 50
33	IPNAT00149a/M01	*Macaranga gigantea*	Mahang Gajah	Methanol	No; 0.937 ± 0.20	> 50
34	IPNAT00142a/M01	*Mallotus barbatus*	Balik Angin	Methanol	No; 0.693 ± 0.22	> 50
35	IPNAT00126a/M01	*Elateriospermum tapos*	Pokok Perah	Methanol	No; 1.136 ± 0.07	> 50
36	IPNAT00119a/M01	*Manihot esculenta*	Ubi Kayu	Methanol	No; 0.724 ± 0.15	> 50
37	IPNAT00145a/M01	*Croton argyratus*	Hujan Panas	Methanol	No; 1.233 ± 0.05	> 50
38	IPNAT00167a/M01	*Callerya atropurpurea*	Tulang Daing	Methanol	No; 1.188 ± 0.03	> 50
39	IPNAT00231a/M01	*Lithocarpus rassa*	Mempening	Methanol	No; 0.399 ± 0.23	> 50
40	IPNAT00289a/M01	*Lithocarpus lucidus*	Mempening	Methanol	Yes; 0.415 ± 0.05	> 50
41	IPNAT00288a/M01	*Lithocarpus curtisii*	Mempening	Methanol	Yes; 0.452 ± 0.05	> 50
42	IPNAT00267a/M01	*Hanguana malayana*	Bakong Rimba	Methanol	No; 1.132 ± 0.00	> 50
43	IPNAT00143a/M01	*Vitex quinata*	Halban	Methanol	No; 0.814 ± 0.29	> 50
44	IPNAT00000a/M01	*Tetracera scandens*	Akar Mempelas	Methanol	Yes; 0.386 ± 0.03	13.041 ± 0.82
45	IPNAT00138a/M01	*Cinnamomum porrectum*	Medang Kemangi	Methanol	Yes; 0.410 ± 0.02	15.986 ± 2.63
46	IPNAT00284a/M01	*Litsea grandis*	Medang Lebar Daun	Methanol	No; 0.597 ± 0.29	> 50
47	IPNAT00164a/M01	Barringtonia macrostachya	Putat Hutan	Methanol	Yes; 0.565 ± 0.12	31.590 ± 3.75
48	IPNAT00135a/M01	*Millettia chrysamaryssa*	Akar Tuba	Methanol	No; 1.201 ± 0.04	> 50
49	IPNAT00268a/M01	*Angiopteris evecta*	Paku Gajah	Methanol	No; 1.258 ± 0.08	> 50
50	IPNAT00116a/M01	*Pternandra coerulescens*	Sial Menahun	Methanol	Yes; 0.370 ± 0.02	29.049 ± 4.05
51	IPNAT00196a/M01	*Azadirachta excelsa*	Sentang	Methanol	No; 0.771 ± 0.24	> 50
52	IPNAT00346a/M01	*Ficus* sp.	Ara	Methanol	No; 1.173 ± 0.05	> 50
53	IPNAT00058a/M01	*Artocarpus elasticus*	Terap Nasi	Methanol	No; 0.754 ± 0.04	> 50
54	IPNAT00286a/M01	*Ficus grandiflora*	Ara	Methanol	No; 0.611 ± 0.12	> 50
55	IPNAT00151a/M01	*Ficus superba*	Ara	Methanol	No; 0.755 ± 0.10	> 50
56	IPNAT00154a/M01	*Artocarpus rigidus*	Temponek	Methanol	No; 0.499 ± 0.12	> 50
57	IPNAT00157a/M01	*Streblus elongatus*	Tempinis	Methanol	Yes; 0.398 ± 0.03	31.806 ± 3.16
58	IPNAT00162a/M01	*Ficus fruticosa*	Ara Belukar	Methanol	No; 0.930 ± 0.17	> 50
59	IPNAT00038a/M01	*Averrhoa bilimbi*	Belimbing Buluh	Methanol	No; 1.004 ± 0.09	> 50
60	IPNAT00160a/M01	*Baccaurea motleyana*	Rambai	Methanol	No; 1.083 ± 0.06	> 50
61	IPNAT00281a/M01	*Antidesma* sp.	Totua	Methanol	No; 0.757 ± 0.02	> 50
62	IPNAT00248a/M01	*Baccaurea* sp.	Setambun	Methanol	No; 0.656 ± 0.19	> 50
63	IPNAT00163a/M01	*Antidesma cuspidatum*	Sebasah, Punai	Methanol	Yes; 0.515 ± 0.03	> 50
64	IPNAT00021a/M01	*Piper sarmentosum*	Kaduk	Methanol	No; 0.991 ± 0.21	> 50
65	IPNAT00168a/M01	*Bruguiera cylindrica*	Bakau Putih	Methanol	No; 1.189 ± 0.03	> 50
66	IPNAT00123a/M01	*Gynostroches axillaris*	Mata Keli	Methanol	No; 0.758 ± 0.29	> 50
67	IPNAT00121a/M01	Greenea corymbosa	Tinjau Belukar	Methanol	No; 0.733 ± 0.18	> 50
68	IPNAT00161a/M01	*Nauclea orientalis*	Bangkal	Methanol	Yes; 0.441 ± 0.05	44.843 ± 2.02
69	IPNAT00117a/M01	*Porterandia anisophylla*	Tinjau Belukar	Methanol	No; 0.899 ± 0.21	> 50
70	IPNAT00003a/M01	*Elephantopus scaber*	Rumput Tutup Bumi	Methanol	No; 0.845 ± 0.14	> 50
71	IPNAT00260a/M01	*Maclurodendron porteri*	Merlimau, Rawang	Methanol	No; 1.236 ± 0.09	> 50
72	IPNAT00144a/M01	*Pometia pinnata*	Kasai	Methanol	No; 0.569 ± 0.08	> 50
73	IPNAT00165a/M01	*Erioglossum rubiginosum*	Mertajam	Methanol	No; 1.006 ± 0.06	> 50
74	IPNAT00122a/M01	*Smilax* sp.	Ubi Jaga	Methanol	Yes; 0.265 ± 0.09	> 50
75	IPNAT00443a/M01	*Sterculia parvifolia*	Kelumpang	Methanol	No; 0.426 ± 0.01	> 50
77	IPNAT00092a/M01	*Adinandra dumosa*	Tetiup	Methanol	Yes; 0.384 ± 0.06	14.473 ± 3.07
78	IPNAT00136a/M01	*Gonystylus* sp.	Ramin	Methanol	Yes; 0.488 ± 0.07	> 50
79	IPNAT00110a/M01	*Vitex pubescens*	Leban	Methanol	No; 0.971 ± 0.20	> 50
80	IPNAT00125a/M01	*Amomum lappaceum*	Tepus	Methanol	No; 1.104 ± 0.03	> 50

**Table 4:** Bioactive compounds present in various extracts of *T. scandens* of previous research.

Type of extract or partition	Bioactive compounds	Source
Hexane extract of *T. scandens* leaves	3′-5′-diprenylgenistein; 6,8-diprenylgenistein; Alpinumisoflavone; Astragalin; Derrone; Genistein; Hypolaetin; Isoscutellarein; Kaempferol;Kaempferol-3-O-(6″-O-p-trans-coumaroyl) glucoside; Quercetin	(Rosdi 2018)
Ethyl acetate partition of methanol extract from *T. scandens* branches	3′-5′-diprenylgenistein; 6,8-diprenylgenistein; Alpinumisoflavone; Derrone; Genistein	(Lee *et al.* 2009)
Dichloromethane extract of *T. scandens* leaves	Astragalin; Betulinic acid; Hypolaetin; An isomeric mixture of sitosterol, glycoside and stigmasterol glycoside Isoscutellarein; Kaempferol; Kaempferol-3-O-(6″-O-p-trans-coumaroyl) glucoside; Quercetin; Stigmasterol	(Ahmed *et al.* 2014)
Methanol extract of *T. scandens* stem	28-*O*-β-D-glucopyranosyl ester; Betulinic acid; Emodin; Kaempferol; Platanic acid; Quercetin; Tiliroside	(Nguyen *et al.* 2004)

## APPENDICES

### Appendix A

**One of the trials exhibited the microplate assay’s dynamic range capturing signal, blank and control absorption.**

Figure S1 The legend shows the absorbance reading (y-axis) of mixture versus the mixtures in well numbered 1–4 (x-axis) using ascorbic acid as standard compound. One of the experiments showed the microplate assay’s dynamic range that captured the absorption reading of signal, blank and control. This step is important to normalise results and eliminate false-positive results. Well No.1 contained assay buffer with ethanol; well No. 2 contained assay buffer with standard compound and ethanol; well No. 3 contained assay buffer with DPPH and ethanol and well No.4 contained assay buffer with standard compound, DPPH and ethanol.

### Appendix B

**A 384-well plate used for primary screening. Colour shifting during incubation was scored ‘yes’ or ‘no’.**

Figure S2 An example of a 384-well plate during primary screening. A score of ‘yes’ or ‘no’ in colour changing upon incubation ended. Antioxidative agents in plant extracts reduced DPPH colour from purple to pale yellow. The positive samples were further confirmed by reading the adsorption at A530nm via a multimode plate reader (BioTek®, US). The reading should be below 0.5, as the blank (without samples) reading was around 1.0. A ‘yes’ score was given when both criteria that are colour changes and A_530_ < 0.5 were achieved. The sample was classified as ‘inactive’ (or ‘no’) score when pale purple colour was observed in both wells.

### Appendix C

***Tetracera scandens*** **herbarium, put in the MyNature50000 library by a licensed botanist.**

Figure S3 Herbarium of *Tetracera scandens*, identified by a certified botanist and deposited into the MyNature50000 library.
