# Supplementary figures and images for: Establishment of High-Throughput Screening Assay using 384-Well Plate for Identification of Potent Antioxidants from Malaysian Local Plants Repository and Phytochemical Profile of Tetracera Scandens
Source: Trop Life Sci Res. 2025 Jul 31;36(2):23–57. doi: 10.21315/tlsr2025.36.2.2 (PMC12618087; doi:10.21315/tlsr2025.36.2.2)

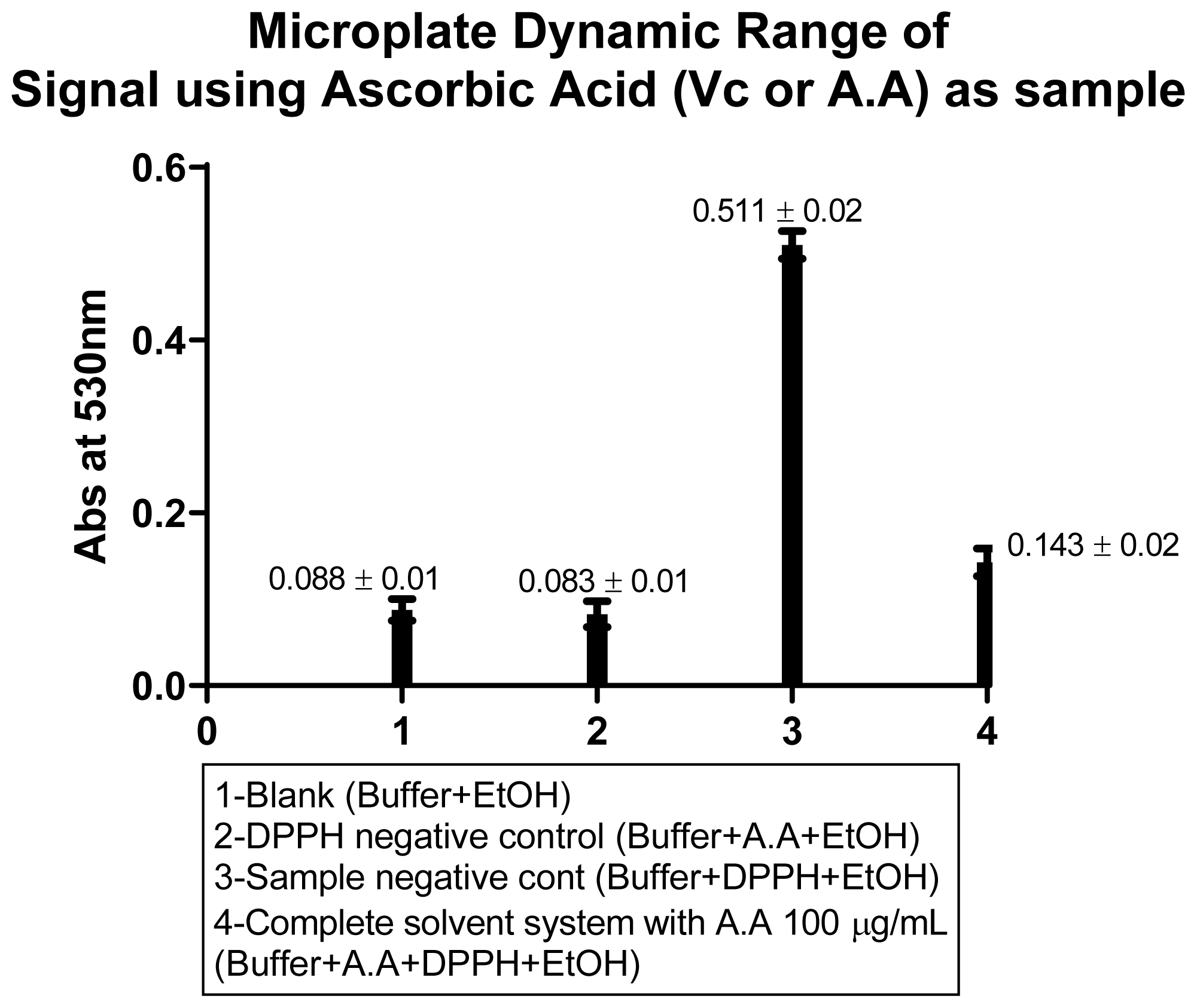

Supplement: Figure S1 — The legend shows the absorbance reading (y-axis) of mixture versus the mixtures in well numbered 1–4 (x-axis) using ascorbic acid as standard compound. One of the experiments showed the microplate assay’s dynamic range that captured the absorption reading of signal, blank and control. This step is important to normalise results and eliminate false-positive results. Well No.1 contained assay buffer with ethanol; well No. 2 contained assay buffer with standard compound and ethanol; well No. 3 contained assay buffer with DPPH and ethanol and well No.4 contained assay buffer with standard compound, DPPH and ethanol. [file tlsr-36-2-23-s001.tif]

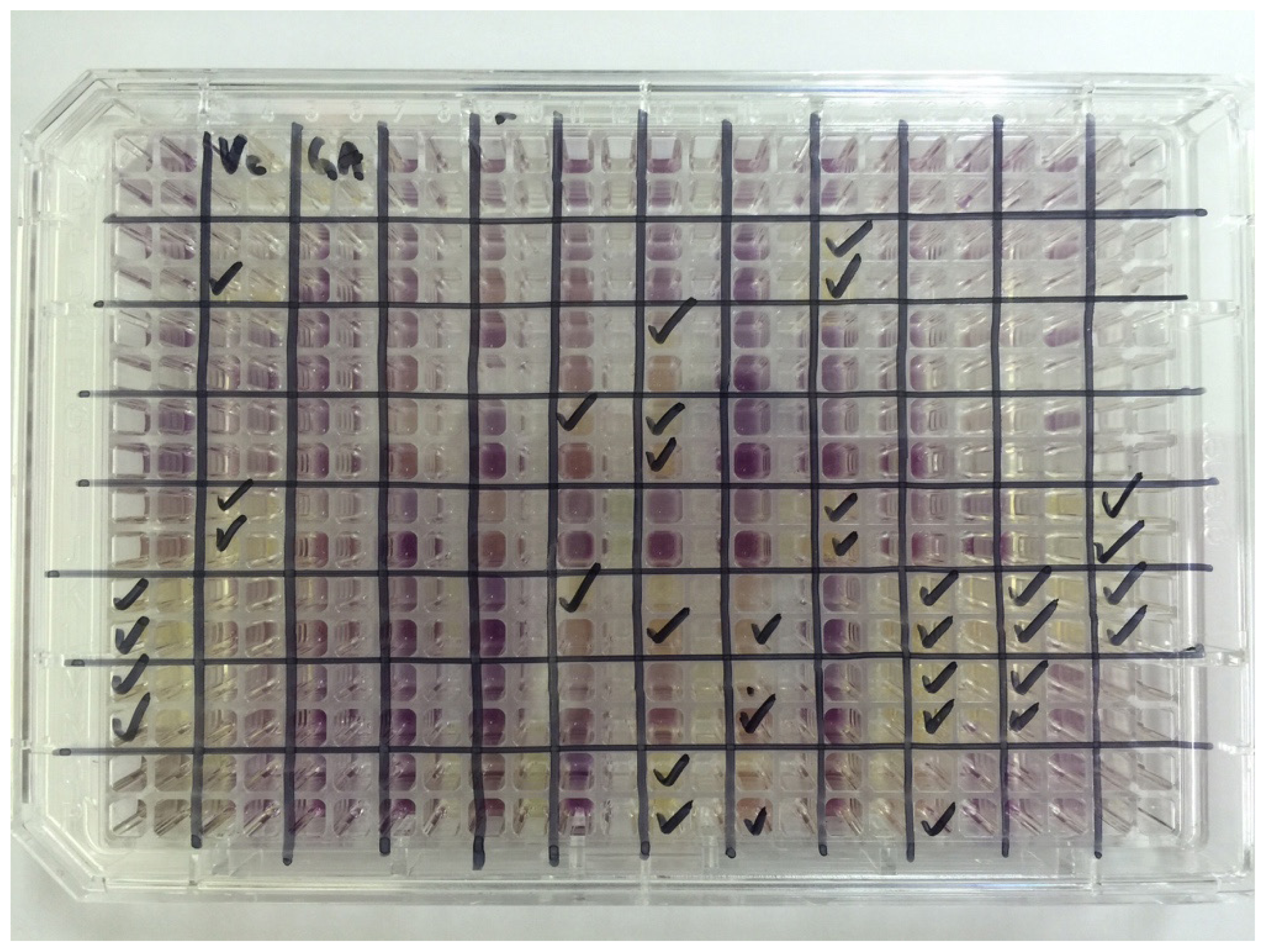

Supplement: Figure S2 — An example of a 384-well plate during primary screening. A score of ‘yes’ or ‘no’ in colour changing upon incubation ended. Antioxidative agents in plant extracts reduced DPPH colour from purple to pale yellow. The positive samples were further confirmed by reading the adsorption at A530nm via a multimode plate reader (BioTek®, US). The reading should be below 0.5, as the blank (without samples) reading was around 1.0. A ‘yes’ score was given when both criteria that are colour changes and A530 < 0.5 were achieved. The sample was classified as ‘inactive’ (or ‘no’) score when pale purple colour was observed in both wells. [file tlsr-36-2-23-s002.tif]

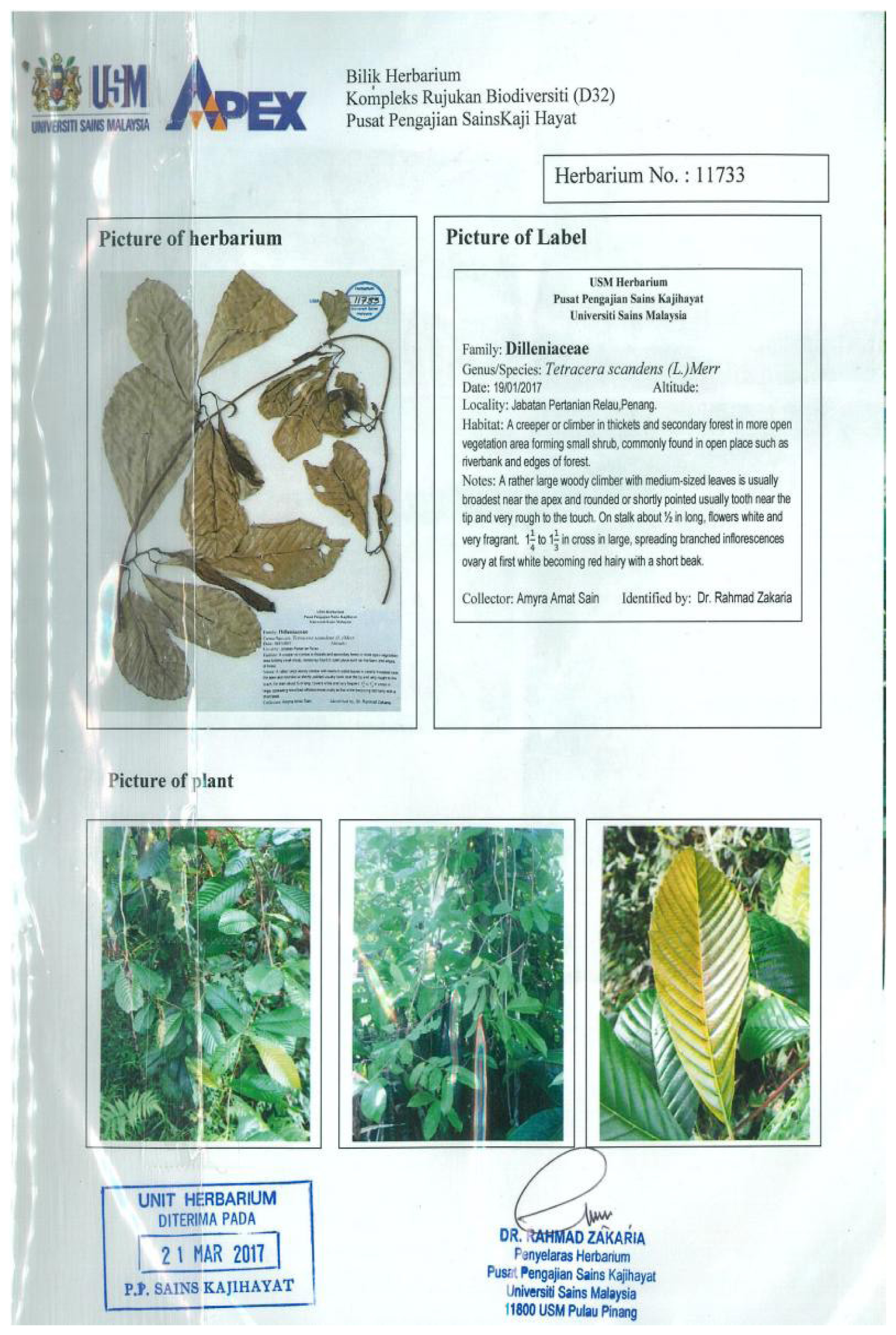

Supplement: Figure S3 — Herbarium of Tetracera scandens, identified by a certified botanist and deposited into the MyNature50000 library. [file tlsr-36-2-23-s003.tif]
